# Queer as F**k: Reaching and Engaging Gay Men in Sexual Health Promotion through Social Networking Sites

**DOI:** 10.2196/jmir.2334

**Published:** 2013-02-07

**Authors:** Alisa Pedrana, Margaret Hellard, Judy Gold, Nadine Ata, Shanton Chang, Steve Howard, Jason Asselin, Olivia Ilic, Colin Batrouney, Mark Stoove

**Affiliations:** ^1^Burnet InstituteCentre for Population HealthMelbourneAustralia; ^2^School of Public Health and Preventive MedicineDepartment of Epidemiology and Preventive MedicineMonash UniversityMelbourneAustralia; ^3^The Nossal Institute for Global HealthThe University of MelbourneMelbourneAustralia; ^4^Faculty of MedicineMonash UniversityMelbourneAustralia; ^5^Melbourne School of EngineeringDepartment of Computing and Information SystemsThe University of MelbourneMelbourneAustralia; ^6^Victorian AIDS Council/Gay Men’s Health CentreHealth Promotion TeamMelbourneAustralia; ^7^X:Machine Productions Pty. Ltd.MelbourneAustralia

**Keywords:** health promotion, Internet, social networking sites, sexual health, gay men

## Abstract

**Background:**

A growing number of health promotion interventions are taking advantage of the popularity and interactivity of new social media platforms to foster and engage communities for health promotion. However, few health promotion interventions using social networking sites (SNS) have been rigorously evaluated. "Queer as F**k"(QAF) began as pilot project in 2010 to deliver sexual health promotion via short "webisodes" on SNS to gay men. Now in its fifth season, QAF is among the few published examples internationally to demonstrate the sexual health promotion potential of SNS.

**Objective:**

The objective of this evaluation is to assess reach, interactivity, and engagement generated by QAF to inform future health interventions and evaluations using SNS.

**Methods:**

We undertook a mixed method process evaluation using an uncontrolled longitudinal study design that compared multiple measurements over time to assess changes in reach and engagement. We adapted evaluation methods from the health promotion, information systems, and creative spheres. We incorporated online usage statistics, interviews informed by user diary-scrapbooks, and user focus groups to assess intervention reach and engagement.

**Results:**

During Series 1-3 (April 2010 to April 2011), 32 webisodes were posted on the QAF Facebook and YouTube pages. These webisodes attracted over 30,000 views; ranging from 124-3092 views per individual episode. By April 2011, the QAF Facebook page had 2929 predominantly male fans. Interview and focus group participants supported the balance of education and entertainment. They endorsed the narrative "soap opera" format as an effective way to deliver sexual health messages in an engaging, informative, and accessible manner that encouraged online peer discussion of sexual health and promoted community engagement.

**Conclusions:**

QAF offers a successful example of exploiting the reach, interactivity, and engagement potential of SNS; findings from this process evaluation provide a model to inform the delivery and evaluation of future health promotion interventions on SNS.

## Introduction

### Background

The Internet is increasingly recognized as a platform for health communication and education due to its enormous and growing reach and ability to share information unrestricted by geographical location and time [1-3]. The advent of social media and Web 2.0 applications like social networking sites (SNS), blogs, wikis, podcasts, RSS feeds, and online support groups have revolutionized Internet use and dramatically changed the nature of online engagement and the cumulative time individuals spend communicating, interacting, and accessing information. Eager to capitalize on this potential, many organizations have developed online health interventions for a variety of health issues and clinical outcomes [1,4-6], including for HIV prevention to gay men [7-9] with some reporting positive outcomes [10-12]. Yet to date, there have been very few published examples of evaluation of interventions delivered on SNS [13]; a recent review of sexual health promotion on SNS found the vast majority of activities are unreported in the scientific literature and showed limited success in practice [14]. One very recent randomized controlled study in the United States showed some promising results for SNS as a sexual health education tool [15]. This trial aimed to determine the effect of STI prevention messages delivered to youth via Facebook in reducing sexual risk behaviors compared to a control group that received news stories via Facebook. Findings showed mild effects for condom use (intervention 68% vs control 56%, *P*=.04) and proportion of sex acts protected by condoms (intervention 63% vs control 57%, *P*=.03) at 2-month follow-up; however, no lasting effects were reported at 6 months.

SNS are of particular interest for health promotion due to their enormous potential audience reach and interactive features. SNS allow individuals to create online “profiles” and connect with other users within the system [16]. SNS act as an “open communication” channel to foster social interactions, create online communities [17], and allow the sharing of user-generated content [16]. Previous studies have shown benefits of such interactive health communication capabilities to enhance learning [18]. The adaptive and interactive features of Web 2.0 applications like SNS that allow increased user-generated content have the potential to promote active and engaged learning [19], whereby users “construct their own knowledge through social interaction and exploration” [20]. By encouraging communication between users or creating “community dialogue”, SNS have the potential to encourage active learning, as well as peer-to-peer learning. These learning strategies have shown some potential in helping individuals internalize and process messages and increase knowledge and improve attitudes and skills for HIV prevention and sexual health; however, these data are largely inconclusive [21-23]. Additionally, using social networking features, interventions are able to disseminate health messages quicker through a population when compared to traditional forms of social marketing [24]. Yet to date, no study has assessed the relative effect of novel functions of SNS for health promotion, and these dimensions present obvious challenges for both monitoring and evaluating impact. Additional dimensions to evaluate SNS-based health promotion not typically considered in traditional media approaches, such as user interactions, functions to support interaction, content quality, and credibility of content have been suggested as useful tools to help evaluation future interventions in this space [20].

In 2010, we launched “Queer as F**k” (QAF), an innovative and novel sexual health promotion intervention using SNS to target gay men in Victoria, Australia [25,26]. We have previously published implementation recommendations based on our experience in the first phase of this project targeting young people [27]; this paper reports the results of the process evaluation of QAF over the initial pilot phase (Series 1) and through the subsequent two series. The aim of this evaluation is to assess reach, interactivity, and engagement generated by QAF to inform future health interventions and evaluations using SNS.

### The Queer as F**K Project

**Figure 1:**
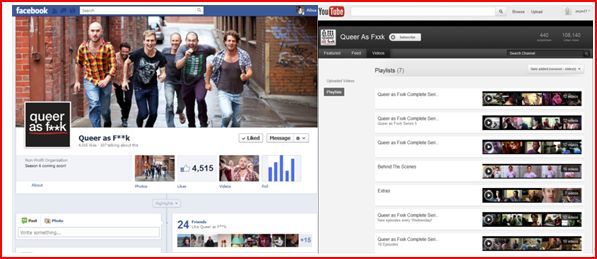
Screen shots of Facebook and YouTube.

**Figure 2:**
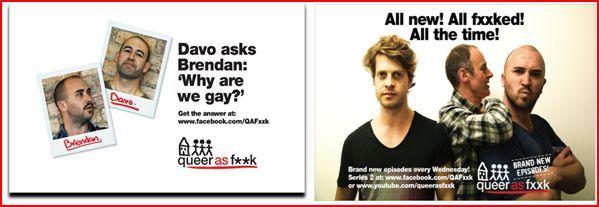
Press ads in gay community magazine used for promotion of QAF project.

QAF originated as one arm of “The FaceSpace Project”, which tested the delivery of sexual health promotion via SNS to two key at-risk groups: young people aged 16-29 years and gay men in Victoria, Australia [27]. QAF was designed as a drama series featuring 4 fictional gay characters, with health messages delivered through short “webisodes” posted on Facebook and YouTube (see Figure 1), and in accompanying online narrative. The narratives and health messages were developed during formative evaluation workshops with members of the target audience and key stakeholders. Much like a TV drama, this online drama series was made up of sequential and individually discrete series, each containing a number of webisodes. Series 1 included 10 webisodes posted on the QAF Facebook and YouTube pages from April 12 - August 10, 2010. Series 2 contained 12 webisodes posted from October 5 - December 21, 2010, and Series 3 consisted of 12 webisodes posted from February 16 - April 18, 2011. Between episodes, project staff posted questions and content daily on the QAF Facebook page to prompt online discussion about the sexual health issues embedded in the narrative of the QAF webisodes and encourage interaction with and between QAF Facebook fans. QAF was promoted through a mix of online and offline advertising, including press advertisements (Figure 2) and editorial coverage in local gay media, Facebook advertisements, updates to fans through the QAF page, and community engagement at gay public events.

The project was a collaboration between public health researchers (Centre for Population Health, Burnet Institute), experts in user interaction with information technologies (Department of Computing & Information Systems, University of Melbourne), a creative productions company (X:Machine), and a community organization with marketing and production expertise (Victorian AIDS Council/Gay Men’s Health Centre, VAC/GMHC).

The primary aims of QAF were to: (1) explore the extent to which SNS can reach and engage gay and bisexual men and improve their knowledge and attitudes to sexual health, and (2) provide recommendations of appropriate frameworks for evaluating health promotion interventions delivered via SNS.

Following the relative success of Series 1, funding to continue QAF was secured. Over the first three series, sexual health promotion topics covered by QAF included sero-discordant relationships, unprotected sex, post-exposure prophylaxis (PEP) [28], strategic positioning [29], sexual health testing, coming out to family, casual hook-ups, HIV disclosure, sero-conversion, alcohol and recreational drugs, and surrogacy.

## Methods

### Overview

To monitor and evaluate QAF, we undertook a mixed method process evaluation using an uncontrolled longitudinal study design comparing multiple measures over time to assess changes in reach and engagement. We adapted and combined evaluation methods from the health promotion (eg, focus groups), information systems (eg, usage statistics), and creative spheres (eg, creative/development workshops) to create a dynamic and appropriate evaluation framework (Multimedia Appendix 1) [20]. Project reach (who we were able to recruit) was measured, as was the level of engagement and interaction (degree of fan interest and interaction) using repeat measures over time of website insight statistics, a qualitative diary, and focus groups. An iterative approach allowed QAF evaluation findings to feed back into the project implementation and evaluation, with knowledge gained from previous phases used to improve intervention delivery. This paper describes process evaluation outcomes from Series 1-3. Evaluation periods were constructed around the three series implementation dates (Series 1 = April 1 - August 31, 2010; Series 2 = September 1 - December 30, 2010; Series 3 = January 1 - April 30, 2011), and data were compared across the three time periods.

### Data Collection Methods and Analysis

#### Website Insight Statistics (Series 1-3)

Insight statistics were downloaded from Facebook and YouTube on a weekly basis, monitored throughout the project, and used to measure reach, engagement, and interaction. Facebook data included fan demographics (gender, age group, country where fan is based), usage data (unique page views, active users, photo views), and total interactions (wall posts, comments, “likes” per day). Fans refer to people who “like” a Facebook page. A user was considered “active” by Facebook if they viewed or engaged with the QAF page or any content on the page. YouTube data included cumulative number of video views, demographics, and traffic sources, which described where users accessed the YouTube channel from. However, Youtube demographics data were available only for logged-in users. The number and proportion of logged-in users compared to total users was unreported and thus unknown, yet was thought to be only a small proportion [30].

Descriptive analysis of insight statistics assessed reach, delivery, and engagement for the three evaluation periods, and data are presented individually for all three series and then compared between Series 1 and Series 2-3 combined.

#### Diary Scrapbook Activity (Series 1 Only)

A qualitative diary scrapbook activity was chosen to collect prospective data on engagement and interaction of fans with the project page and reduce recall bias and improve data validity by providing real-time information [31-33]. The aim was to provide information surrounding the context of engagement with QAF and identify potential drivers of participant engagement for future QAF series. Recruitment occurred through an online quantitative survey, which was advertised during Series 1 to all fans of the QAF fan page by posting a link to the survey on the wall of the QAF Facebook fan page and via Facebook advertisements; only 188 (14.2%) participants completed a baseline survey. Survey data revealed very few meaningful insights to inform the project or measure impact, thus these data are not reported here. Participants who completed an online survey and agreed to participate further in evaluations were then invited via email to participate in the diary-scrapbook activity. Participants then attended a face-to-face introductory briefing, received their diary scrapbook, and signed a participant and information consent form. The diary scrapbook activity aimed to gain information about participant engagement with QAF and involved participants regularly recording their weekly activity on the QAF sites in a diary-scrapbook for 6 weeks (June 8^th^ - July 20^th^, 2010). After 6 weeks, participants returned their diary scrapbook via regular mail and participated in a follow-up interview in which diary-scrapbook content was used to guide the interview. Interviews were conducted face-to-face, took between 30 and 50 minutes, and were audio recorded. Participants were reimbursed AUD$100 in cash for participation in the diary-scrapbook activity. The diary-scrapbook follow-up interviews were thematically analyzed to assess and contextualize participant engagement with QAF. Of the 10 men who agreed to participate in the diary-scrapbook activity, 9 completed the activity and interview. Participants’ age ranged from 27 to 47 years (medium 38 years). Results for the diary-scrapbook activity interviews are presented only for Series 1 and presented together with Series 1 focus group data.

#### Focus Groups (Series 1-3)

To support website usage data, we conducted a series of qualitative focus groups to provide more in-depth information on engagement and interaction through Series 1-3 and explore the perceived utility of QAF and SNS more generally for sexual health promotion. Four evaluation focus groups were conducted; two at the end of Series 1 (November 2010), and one each at the end of Series 2 (January 2011) and Series 3 (May 2011). Focus group participants were recruited from the pool of online survey participants who had agreed to participate in further evaluation. Focus group schedules included themes regarding general uses of SNS, reflections on QAF (aims, content, website layout, and characters), strategies to drive interaction, and future improvements. Focus group participants were reimbursed AUD$50 in cash for time and traveling costs. All focus group data were audio recorded and transcribed. Transcripts were analyzed thematically to assess participant engagement with QAF [34-36]. Focus group data are presented by series and then compared between Series 1 and Series 2-3 combined. Fourteen participants attended two Series 1 focus groups. Participants’ age ranged from 21 to 46 years (medium 35 years). Thirteen participants attended two Series 2 and 3 focus groups. Participants’ age ranged from 22 to 46 years (medium 34 years).

Qualitative results from both the diary scrapbook and focus groups are presented together under “Reach” and “Engagement and Interaction”, as emerging themes and findings were largely shared by both groups. However, under “Engagement and Interaction”, data are divided into three themes: 1) participant engagement and interaction with the QAF project, 2) participant engagement and interaction with the sexual health content of the QAF project, and 3) barriers to participant engagement and interaction with QAF project.

### Ethics

Ethics approval for this project was obtained from the Alfred Health Human Ethics Committee.

## Results

### Series 1 (Pilot): Trailing the Approach

#### Reach

##### Facebook Insight Statistics

**Figure 3:**
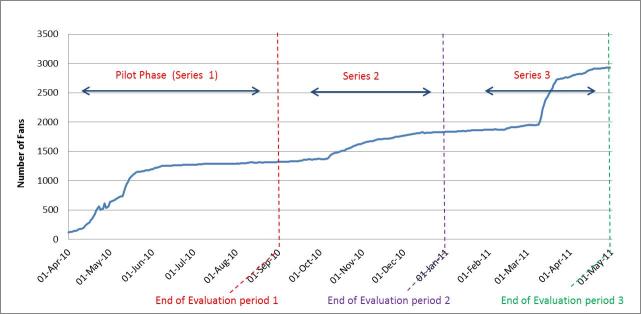
Total fans over time, on QAF Facebook page from Series 1-3.

At the end of the Series 1, QAF had reached 1320 fans. The majority of fans were male (80%) and based in Australia (87%) (Table 1). Around two thirds of male fans were aged 25-44 years (Table 1). There was a rapid increase in number of fans in April-May 2010, coinciding with initial series promotion (Figure 3). The greatest increase in numbers of fans (from 782 to 1153) coincided with the use of Facebook advertisements (May 11 – 21, 2010) (Figure 3).

##### YouTube Insight Statistics

At the end of Series 1, the QAF YouTube Channel had received 7297 video views. The majority of logged-in viewers were male (92%), located in Australia (72%), and aged 44-54 years (43%) (Table 2). The most popular video was Episode 1, “I’ve Never...Felched”, which covered coming out and past sexual experiences, with 1672 video views. The next most viewed was Episode 2, “Lady Gaga on a Disco Stick”, which covered drug use and risky sex, with 1077 views.

**Table 1:** Key metrics from the QAF Facebook page usage statistics per series (source: Facebook insights statistics).

Variables			Series 1	Series 2	Series 3
Evaluation period			April – Aug. 2010	Sept. – Dec 2010	Jan. - April 2011
**Reach**					
	Total fans at series conclusion (cumulative)		1320	1835	2929
	Number of new fans reached		1199	501	1094
	n (%) male		1026 (80.3)	1446 (81.5)	2424 (84.7)
	Age groups, n (%)				
		13-17 years	54 (2.2)	34 (2.4)	54 (2.2)
		18-24 years	641 (19.1)	272 (18.8)	641 (26.4)
		25-34 years	784 (33.0)	500 (34.6)	784 (32.3)
		35-44 years	582 (29.8)	399 (27.6)	582 (24.0)
		45-54 years	275 (11.9)	184 (12.8)	275 (11.3)
		> 55 years	88 (3.9)	57 (3.9)	88 (3.6)
	Top countries where fans are based, n (%)				
		Australia	1115 (87.4)	1493 (85.3)	2504 (88.4)
		United States	44 (3.5)	75 (4.3)	107 (3.8)
		United Kingdom	41 (3.2)	76 (4.3)	91 (3.2)
		Other	76 (5.9)	57 (6.1)	132 (4.6)
**Engagement and Interaction**					
	Total page interactions		526	942	927
		Likes	281	546	495
		Comments	205	380	413
		Wall posts	40	16	19
	Unsubscribes		39	24	10
	Unique page views		6105	4898	5771
	Video views		2642	9608	9903

**Table 2:** Key metrics from the QAF YouTube page usage statistics per series (source: YouTube insights statistics).

Variables			Series 1	Series 2	Series 3
Evaluation period			April – Aug. 2010	Sept. – Dec. 2010	Jan. - April 2011
**Reach**					
	Total video views per series		7297	9594	14466
	Number of views of most popular episode		1672	831	1816
	Proportion male (%)^a^		92.1	91.2	91.1
	Age group of male fans (%)^a,b^				
		13-17 years	0.0	0.0	0.8
		18-24 years	6.1	7.3	4.3
		25-34 years	8.6	12.6	7.9
		35-44 years	21.1	22.3	25.3
		45-54 years	43.0	29.4	37.5
		> 55 years	13.3	19.5	15.3
	Top countries where fans are based (%)^a^				
		Australia	71.6	73.2	67.2
		United States	13.7	8.3	9.8
		United Kingdom	2.4	3.0	4.2
		Saudi Arabia^c^	-	2.2	4.4
**Engagement and Interaction**					
	New subscribers		79	61	44
	Likes		36	70	75
	Dislikes		1	0	3
	Favorites		17	15	23
	Comments		11	9	17
	Sharing		0	0	5

^a^These variables are based on the number of users with a YouTube profile, which account for only a small proportion of the total sample, thus caution should be taken when interpreting these results.

^b^Percentages of male fans only; so do not add up to 100%.

^c^In Series 3, Saudi Arabia took over the United Kingdom as the third country with most video views.

##### Diary Scrapbook Activity and Focus Groups

The majority of participants reported first finding out about QAF from Facebook advertisements. Facebook advertisements appeared to be more effective at attracting fans than any other form of promotional materials, including advisements in gay media (Figure 2); although a few people became aware of the project through their online social networks: “I came in at Episode 4, a friend shared it with me on Facebook, and then I got hooked!” (focus group participant). Some participants described barriers to project reach, which were largely focused around the limitations of the medium/platform in which the intervention was delivered, reporting that they either did not notice the QAF project’s presence on Facebook among the other traffic or did notice the QAF project but their attention was quickly directed to one of the many other activities on Facebook: “There’s so much stuff [on Facebook] that is released all the time … even if you design something really good, it’s released into this huge noise of material that’s released every day, every hour” (diary participant) and “With 300 odd friends on your list [on Facebook], the posts [on Facebook] go through very fast … so you don’t always get to see it” (diary participant).

#### Engagement and Interaction

##### Facebook Insight Statistics

**Figure 4:**
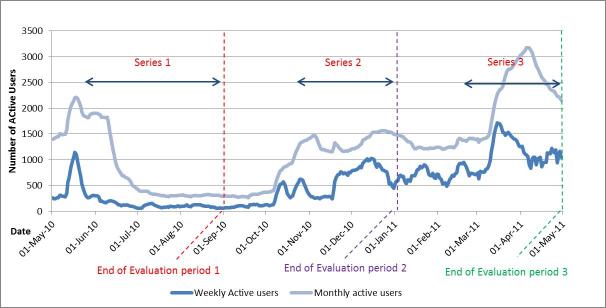
Total number of weekly and monthly active fans, on QAF Facebook page from Series 1-3.

**Figure 5:**
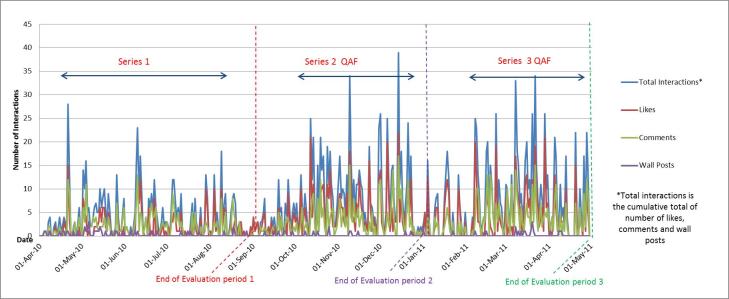
Total number of unique page interactions (includes wall posts, comments, and ‘likes’) over time, on QAF Facebook page from Series 1-3.

At the end of Series 1, the QAF page had received 6105 unique page views, 2642 individual video views, and 526 page interactions, including 281 likes, 205 comments, and 40 wall posts (Table 1). There were peaks in active users and unique pages views during the early stages of implementation, particularly during the initial promotion period (April-May 2010), then a plateau throughout the rest of the series (Figure 4). The peaks in page interactions coincided with webisode postings (Figure 5).

##### YouTube Insight Statistics

The QAF YouTube channel had received 7297 video views by the end of Series 1, which, along with the 79 subscriptions to the channel, 36 likes, and a small proportion of comments and favorites the page received, provided evidence of user engagement and interaction (Table 2).The comments from fans were largely positive and included quotes such as “Very cute! That scene would be really funny if they were all drinking VB [beer]”, “OMG! I love this parody!”, and “Very funny”.

#### Diary Scrapbook Activity and Focus Groups

##### Participant Engagement and Interaction With the QAF Project

Participants reported the main reason for visiting and interacting with the QAF page was to watch the webisodes. They described the webisodes as interesting and engaging and liked the interactive web-based soap-opera style. They also commented positively on the quality of the content produced: “I found the videos really interesting and the videos were well produced ... that was the thing that drove me to go back [to the page] a few times” (diary participant), “I went back just to watch the videos, I found them really good” (diary participant), and:

I felt comfortable watching it, it was entertaining. I didn’t get the sexual health message at first, it’s quite subtle. Entertaining to watch which kept my interest.

focus group participant

##### Participant Engagement and Interaction With the Sexual Health Content of the QAF project

Participants also describe how they liked the subtlety and realism of the content of the webisodes. They also discussed how the style of the presentation of the sexual health information (via video), made them feel comfortable to engage with the project:

I mean [the episodes] are digestible, they’re good. They show a true side to gay friends

getting together and talking about probably what some people would see as trivial things in our lives, but they’re actually real things in our lives.

diary participant

I like it because I’m comfortable watching it. But I’m also uncomfortable at times. There are certain episodes that break me, but I can relate to those episodes because I know people who would react like that.

focus group participant

I respond better and like it because it’s subtle, it’s not rammed down your throat. I wouldn’t respond well if it was rammed down my throat.

focus group participant

Some participants also discussed how they thought QAF provided the gay community with an opportunity to discuss sexual health content on Facebook with peers, which was a positive step: “I think congratulations, it’s a really useful tool for the community that allows them to interact, and talk about subjects that I don’t think really exist so there’s a real need for it” (diary participant).

##### Barriers to Participant Engagement and Interaction With the QAF Project

Although most participants expressed initial enthusiasm for the project, they described that after a few episode they tended to lose interest. There were two main reasons cited for not returning to the QAF Facebook page. First, the QAF Facebook page was not very visible in the large amount of traffic occurring on their Facebook newsfeeds. Second, the frequency of webisode posts without sufficiently engaging additional content on the QAF page was a barrier to coming back:

The long wait between episodes and the length…it’s easy to forget about the project. And there was almost no reason to go back … I probably watched two or three times and that was it …

focus group participant

When I first signed up to it, I probably went on two or three times in that first week and then

it was just like totally forgotten about.

focus group participant

Participants also reported that the public nature of Facebook meant they were careful about what they commented on because it would show up in their status updates. Some participants questioned the suitability/appropriateness of Facebook as a forum for discussing sexual health: “Maybe it was a bit odd, talking about [sexual health issues] on Facebook…it’s not really the right forum, like you’re not in the mind space to be talking about this kind of stuff” (diary participant) and “I didn’t really even necessarily have a reason to interact. I don’t know why ... The way [QAF] is doesn’t really seem like a social site” (diary participant).

I don’t know that Facebook lends itself to sexual health promotion in some ways … I tend to engage on Facebook as a communicative method to keep in touch with my friends. But there are interests and causes that I like pages for … like a justice cause ... I can’t see how sexual health fits into either of those.

focus group participant

Some participants acknowledged that their usual habits or interactions on Facebook dictated the extent of their engagement with the QAF page, not necessarily the content of the project: “I tend not to comment a lot on Facebook anyway. The only times I tend to comment on people’s various updates and things is if I know them particularly well” (diary participant) and “Normally I wouldn’t write comments [on Facebook fan pages], I’d normally just ‘like’ something” (diary participant).

### Series 2 and 3 – Increasing Reach and Engagement

Building on the success of the Series 1 pilot and aiming to capitalize on the existing fan base, QAF continued into further series. Several changes were made to the subsequent series based on evaluation findings from Series 1 (Table 3).

**Table 3:** Changes made to QAF project implementation following evaluation of Series 1 pilot.

Challenges from Series 1	Changes for subsequent series
Plateau of new fans reached by mid-season	Introduction of new characters to increase/sustain engagement
Infrequent & irregular timing of episode releases	Twelve episodes, posted every Wednesday at midday; compared to almost every 2 wks on no particular day in Series 1
Decrease in return of fans to pages	Intensified use of Facebook advertisements to target self-identified gay men
Discussions on Facebook about the webisodes or sexual health issues was minimal, communication still largely one-way	Using dramatic themes in episodes to elicit organic user-led discussion about sexual health

#### Reach

##### Facebook Insight Statistics

There was a steady increase in fans throughout Series 2 and a sharp increase mid-way through Series 3 (Figure 3). By the end of Series 2, QAF had gained 501 fans in addition to those from Series 1 (38% increase from Series 1) to total of 1835 fans. By the end of Series 3, this had reached 2929 fans (59% increase from Series 2). Fans continued to be predominantly male. However, the proportion of younger fans increased in Series 3 (Table 1). While fans remained predominantly based in Australia, by the end of Series 3, there were QAF fans in over 18 other countries. The total number of video views increased dramatically over Series 2 and 3 compared to the first series.

##### YouTube Insight Statistics

At the conclusion of Series 3, the QAF YouTube channel had received 31,357 video views. Compared to Series 1, QAF increased its video views in both series: 9594 views (31% increase) by the end of Series 2 and 14,466 views (98% increase) by the end of Series 3. YouTube viewers (logged-in viewers) remained predominantly male, resided in Australia, and were older at 35-54 years (65%) compared to Facebook fans (Table 2). The QAF YouTube channel received almost a third of their views from other countries; including the United States, United Kingdom, and Saudi Arabia (Table 2).The most popular videos were those of Series 1, with the most popular being Episode 5, “Sex text … call Aaron for a good time”, which deals with multiple sex partners and received 3092 individual video views.

#### Engagement and Interaction

##### Facebook Insight Statistics

**Figure 6:**
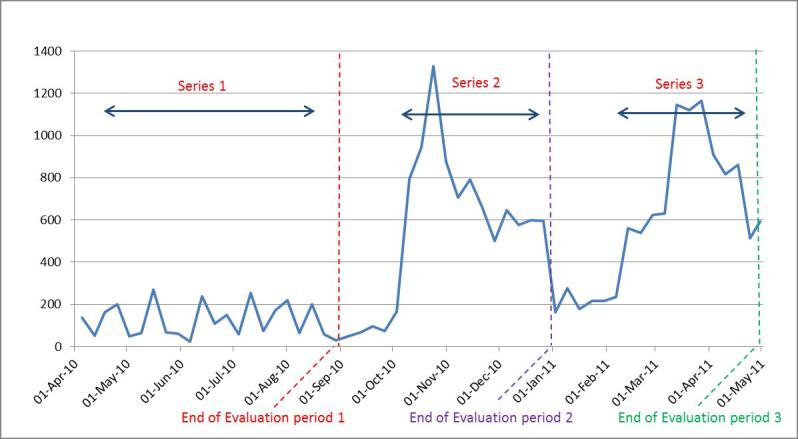
Total number of weekly video views, on QAF Facebook page from Series 1-3.

Compared to Series 1, video views and page interactions, including wall posts, comments, and likes, increased during Series 2 and 3 and displayed a very different dynamic (Figures 5 and 6). Figure 6 clearly demonstrates the increased engagement with the videos over Series 2-3, while Figure 5 shows increased interaction with the page, evident by increases in both wall posts, comments, and likes compared to Series 1. There were similar increases in the proportion of active fans in Series 2 and 3 compared Series 1 (Figure 4). By the second half of Series 3 (Mar.-April 2011), between 50%-70% of fans were active users, interacting with the page at least monthly.

##### YouTube Insight Statistics

By the end of Series 3, the YouTube channel had received over 30,000 video views across all the three series, ranging from 124 to 3092 views per individual episode. Compared to Series 1, the page received increased numbers of fan subscriptions and numbers of likes, comments, and favorites, and by Series 3, fans had begun sharing the QAF videos with their friends, further evidence of user engagement (Table 2). Comments from fans over Series 2-3 progressively went beyond simply endorsing the videos as “funny” as seen in Series 1, and began to discuss and debate the sexual health content of the videos with other users, again evidence of user interaction with both the medium and content. Examples of these comments include: “I don’t know if that’s true. HIV is a big issue, and a big killer, but it’s a surprisingly ineffective virus in terms of infecting people, as in, compared to the common cold etc.”, “HIV isn’t going to infect people at any opportunity ... still important to be safe, but chances of contracting it when practicing safe sex are very low”, and:

I think the HIV+ guy should have been honest straight up, and then let the other guy decide for himself whether he was willing to take the risk. Personally I would still have safe sex with him, but I would most certainly be angered if I was put at risk without my knowledge. It’s not fair on your partner to keep them in the dark about something which could result in their death.

#### Focus Groups

Results from the two focus groups at the end of Series 2 and 3 are presented together, as they covered similar themes. Comments were not necessarily restricted to specific series.

##### Participant Engagement and Interaction With the QAF Project

Similar to Series 1, participants’ engagement and interaction with the QAF project were based around the webisodes. Again participants commented that the high production values and the balance between entertainment and education kept them engaged through subsequent series. Participants also reported that the regular format of posting videos once a week on the same day and the introduction of new characters, including popular celebrities, helped keep them interested and engaged with the project: “It’s good for its entertainment value, and the episodes are short and don’t take long to watch, so don’t have to keep my interest for too long. I liked the subplot with Brendan’s mum, Denise Scott, and her coming to terms with Brendo being gay” (Series 2 focus group participant), “Yep. It gets a bit ridiculous trying to put a message into every episode. Some are just purely entertaining” (Series 2 focus group participant), and “The fact the QAF could fill out a screening at the queer film festival means it must be very engaging” (Series 3 focus group participant).

##### Participant Engagement and Interaction With the Sexual Health Content of the QAF Project

Participants described how they continued to engage and interact with the project throughout subsequent series due to realistic portrayal of the characters and relatable scenarios depicted in the QAF. Participants also were easily able to recall the characters and storylines from the previous series, further evidence of engagement with QAF: “My first impression was that it was an interesting but positive portrayal of gay culture. It had real people in it, people I would know.” (Series 3 focus group participant), “I’ve been in a situation exactly like that, that’s how realistic it was” (Series 2 focus group participant), and:

I think people can relate to Brendan’s vulnerability. And it’s a rational message about PEP – telling people that it needs to be taken within 72 hours, and you have to take it for 28 days. It wasn’t preachy, just realistic.

Series 3 focus group participant

The one with the altercation on the kitchen floor, I hated that one, it clearly sticks in my mind. When he finds out the guy he’s been seeing is positive. It’s most memorable for me.”

Series 3 focus group participant

Some participants also described how QAF provided them with an opportunity to discuss sexual health issues with their peers. They also described how these discussions that occurred on the page with other fans and with the QAF project made them feel involved and “gave them a voice”: “It encourages you to talk about your sex life. I’ve spent years of not talking about my sex life with anybody” (Series 2 focus group participant) and:

I like that I can also share it with my friends, both gay and straight. It opens up communication with people who aren’t necessarily part of the target audience. It’s a good discussion point with friends, everyone has different opinions so it’s great to have a discussion about it.

Series 3 focus group participant

I didn’t know a great deal about PEP, so that episode made me find more information about it and share that with my friends who had never even heard about it. So it provided me with new knowledge. Also that it shows that you can have HIV+ve/-ve relationships and showing these in a ‘normal’ positive light, I think it’s great education in that way. I like the way that was handled.

Series 3 focus group participant

##### Barriers to Participant Engagement and Interaction With QAF Project

Similar to Series 1, some participants were still not comfortable with interacting with the page and preferred to just to view the videos or discussions, while others appear to still be engaged with the project but simply chose not to interact with others on the page:

I just watch it. I don’t read the discussions or comment. I only look when I remember, sometimes I forget about it, maybe it’s not in my face enough.

Series 3 focus group participant

Some episodes that I’ve really enjoyed and engaged with and so I read the discussions. But I’m not at a point where I’ll write on the discussions, I don’t feel comfortable putting my view across with my name and photo there. But I do discuss heavily the big issues that come out of the episodes with friends, for example the one where he comes out about being HIV positive.

Series 2 focus group participant

The public nature of Facebook remained a concern throughout the subsequent series and was a potential barrier for some people to engage/interact with the project:

I think it’s missing a website, there needs to be a website for those who don’t use Facebook or YouTube or who want it to be more private. A website would be easy to access and could be anonymous, that could further engage people.

Series 3 focus group participant

## Discussion

The QAF project is among the first published examples of how to develop, implement, and evaluate an online sexual health promotion intervention on SNS [14]. This process evaluation of a pilot study that developed into a sustained health promotion project demonstrates how an iterative and reflexive approach to health promotion interventions can be applied successfully to new media. While many organizations are using SNS for health promotion, the majority are not effectively exploiting SNS functions to engage their target audiences [14]. With the sustained number of fans and increasing engagement over time, QAF provides a useful model for developing health promotion interventions on SNS.

### Reach

Within a relatively short period, the QAF project managed to reach almost 3000 fans and received over 30,000 videos views. While these numbers may not appear large considering the popularity of SNS [37] and the ability for “viral” spread, when considered against other sexual health promotion activities being delivered on Facebook (median of 327 fans, range 15-111,391) and other SNS [14], it is considerable.

Across the three series, the reach of QAF continued to increase. The most successful promotion tool for reaching potential fans was Facebook advertisements, which enabled targeting of fans by age, geographical location, and sexual orientation (ie, “Interested in” males or females). Mid-way through Series 3, Facebook enhanced the targeting capabilities for their advertisements, enabling the targeting of friends of current fans. This resulted in a substantial boost in fan numbers with no additional effort required by the project team. Furthermore, although promotion efforts were focused locally, viewers from over 50 countries were reached, including a considerable number of Facebook fans and YouTube viewers from the United States, United Kingdom, and Saudi Arabia. This result highlights the huge multijurisdictional potential reach of SNS.

### Engagement and Interaction

A key aim of QAF was to explore the use of SNS as a space for engaging gay men in interactive sexual health promotion. This evaluation showed ongoing and increasing participant engagement with QAF across series, as measured through a variety of methods. Fans engaged primarily with the short webisodes in which health promotional messages were embedded, highlighting the utility of video content in engaging fans but also in delivering health messages. Key reasons for fans returning to the site and continuing to engage with the project included the format (video drama series), the content (realistic, relatable, subtle), the quality (high production values), and the entertainment-education or “Edutainment” approach [38]. These results demonstrate the benefits of interactive health communication to engage users on health topics, particularly on sensitive issues such as sexual health, as evidenced by the rich qualitative data presented in this evaluation. Similar findings have been described elsewhere [18-20]. Furthermore, “Edutainment” has emerged as a popular approach [39,40] for increasing “functional” learning through content that both entertains and educates [38].

These attributes place further emphasis on the quality and credibility of content produced in these spaces, as SNS rely on users’ ability to assess the usefulness, utility, and trustworthiness of content before they choose to engage [20]. Throughout this evaluation, qualitative data highlighted how participant engagement and interaction were heavily dependent on the credibility of the video content. This evaluation provided some evidence of the importance of these attributes, as participants consistently described the high production quality of the webisodes, in combination with the realistic characters and storylines as key factors in maintaining their engagement. Participants also described how the QAF Facebook page provided both impetus and space for online discussions with peers and encouraged interactions between fans. Project staff (data not reported here) also noted that by the end of Series 3, less promoting and probing were required by project staff as fans began initiating discussions and debate around sexual health topics and ongoing user-led discussions became more routine. Given the volume of content produced on SNS, intervention designers must carefully consider the quality and credibility of content if they are to be successful in reaching and engaging their audience in a sustained manner. Other similar studies have used different methods for building community engagement through online social networks, with a US-based study choosing to pay community members rather than research project staff to engage their peers in HIV prevention efforts [15].

This evaluation exposed a number of potential barriers to fan engagement. Concerns about privacy and the public nature of Facebook inhibited some people from engaging with the project. Privacy has been identified previously [16,41] as a key barrier to engaging groups in an online environment, particularly on a SNS where there is a lack of anonymity and limited capacity to provide confidentiality for participants. One important consideration here is the different opportunity that SNS can provide for different “types of users” (ie, The Creator, The Critic, The Spectator)[42-44] to engage in a way that is comfortable to them. More in-depth evaluation designs, including more detailed content analyses of discussions that occurred on the QAF page, may offer insights to understand the characteristics and online behaviors of different types of users. Disaggregating analyses of outcome data may also help determine what effects the depth of user engagement may have on overall impact of the intervention. For example, are fans that interact at a high frequency more likely to modify behavior or have greater awareness of the health issue than other fans?

The success of SNS and other online applications to provide opportunities for online communities to form, often created through shared beliefs and values, has resulted in enthusiastic socializing and network building [44]. Exploiting the functionalities of SNS to increase engagement with interventions and excite social activity around topics such as sexual health is an important step to potentially enhance the impact of such interventions on behavioral and attitudinal change [45].

### Evaluation Learnings

A number of key evaluation learnings emerged from this project. The combination of different evaluation methodologies (usage statistics, diary-scrapbook activity, focus groups) provided a rich mix of quantitative and qualitative data enabling assessment of reach, interaction, and engagement. The SNS platform enabled close monitoring of user interaction with QAF via website insight statistics, which included common website usage metrics (ie, number of fans, likes, comments, wall posts, and shares, including changes over time). As suggested by Bennet and Glasgow (2009) [3], it is vital that researchers working in this area develop a key set of metrics for the monitoring of social media and SNS. A recent report by Gordon (2011) [46] suggests a simple framework for planning and reporting social media metrics—SEE, SAY, FEEL, DO—which categorizes website usage metrics into meaningful groups to help plan and measure combination campaigns. With the focus of many publically funded health promotion interventions on impact, one important challenge for future evaluations will be to ensure that enough emphasis is placed on the importance of process evaluation. A detailed process evaluation was crucial for the success of QAF and provided detailed understanding of the key elements of the intervention and the SNS platform that drove reach and engagement with the intervention.

### Limitations

There are several limitations to this evaluation. Usage data were not always complete nor provided as raw data, thus limiting further data manipulation, analysis, or comparisons across SNS platforms or across other interventions. There were only small numbers of participants in the diary-scrapbook activity and the focus groups, and those that chose to participate may be fans more engaged with QAF. Finally, given the limited resources available to implement and evaluate QAF, a detailed qualitative content analysis of the QAF Facebook page for Series 1 to 3 was not feasible. This also limited our ability to undertake an analysis of how learnings can be passed on through people’s online social networks. Such data would provide further insights regarding enhancing participant engagement and reach for such sexual health programs through SNS. However, such an evaluation is planned for subsequent series now that additional evaluation funding has been secured.

### Conclusions

“Queer as F**k” is one of the first published examples of how to develop, implement, and evaluate an online intervention delivering sexual health promotion on SNS. QAF reached a substantial number of fans over a sustained period and continued to increase reach and user engagement and interaction over time. An iterative approach to project development, implementation, and evaluation allowed ongoing improvements to project delivery and expanded reach and engagement to gay men in these important social networking spaces.

## Multimedia Appendix 1

Development and evaluation framework for the QAF Project.

## Abbreviations

PEP: post-exposure prophylaxis
QAF: Queer as F**k
SNS: social networking sites
VAC/GMHC: Victorian AIDS Council/Gay Men's Health Center
