# Supplementary material for: Queer as F**k: Reaching and Engaging Gay Men in Sexual Health Promotion through Social Networking Sites
Source: J Med Internet Res. 2013 Feb 7;15(2):e25. doi: 10.2196/jmir.2334 (PMC3636214; doi:10.2196/jmir.2334)
Supplement: Supplementary file 1 [file jmir_v15i2e25_app1.pdf]

Appendix 1. Development and evaluation framework for the QAF Project.

Development Stages Description	Evaluation Stage	Data Collection Method
1. **Planning and preparation**<br>• Forming and discussion among project team and advisory committee<br>• Obtaining ethics approval<br>• Devising project design, implementation, and evaluation plans<br>• Incorporating learnings from youth arm<br>2. **Character and narrative development workshops**<br>• Conducting workshops & focus groups with target audience, project staff, and key stakeholders<br>• Developing scripts, including characters, narrative, and health messages<br>3. **Creative and online production**<br>• Rehearsing with actors<br>• Filming and editing of the webisodes<br>• Building online sites	**Formative Phase**<br><br>**Evaluation Questions:**<br>➢ Are we developing this in the right way?<br>➢ Are we delivering this in the right way?	➢ Feedback from youth arm<br>➢ Creative/Development workshops<br>➢ Interactive computer laboratory testing (series one only)<br>➢ Project team meetings
4. **Series launch and promotion**<br><br>Promotions included:<br>• Press ads in gay magazines (Figure 2)<br>• Editorial coverage in gay	**Process Phase**<br><br>**Evaluation Questions:**<br>➢ Whom did we reach?<br>➢ To what extent did we reach our target	➢ Website insight statistics (Facebook, YouTube)

media<br>• Targeted online advertising (Facebook advertisements)<br>• Facebook updates to QAF fans<br>• Promotion at gay public events	audience?<br>➢ What promotional methods were most effective for reaching out target audience?	➢ Diary Scrapbook Activity (Series 1 only)<br>➢ Focus groups
5. **Intervention implementation 'live period'**<br>• 10-12 short webisodes per series uploaded onto the Facebook and YouTube sites (Figure 1)<br>• Project staff used prompt questions to encourage project fans to interact with the project pages.<br>• Projects staff utilized functionality of SNS (comments, polls, and links) to further encourage fans interaction.	**Process Phase**<br>**Evaluation Questions:**<br>➢ To what extent did we engage our target audience?<br>➢ What impact (if any) did we have on improving the gay men's knowledge and awareness of sexual health?	➢ Website Insight statistics (Facebook, YouTube)<br>➢ Diary Scrapbook Activity (Series one only)<br>➢ Focus groups
